# A retrospective comparison of active surveillance to stereotactic radiosurgery for the management of elderly patients with an incidental meningioma

**DOI:** 10.1007/s00701-025-06452-4

**Published:** 2025-02-06

**Authors:** Hana Hallak, Georgios Mantziaris, Stylianos Pikis, Abdurrahman I. Islim, Selcuk Peker, Yavuz Samanci, Ahmed M. Nabeel, Wael A. Reda, Sameh R. Tawadros, Amr M. N. El-Shehaby, Khaled Abdelkarim, Reem M. Emad, David Mathieu, Cheng-Chia Lee, Roman Liscak, Roberto Martinez Alvarez, Douglas Kondziolka, Manjul Tripathi, Herwin Speckter, Greg N. Bowden, Ronald J. Benveniste, Lawrence Dade Lunsford, Michael D. Jenkinson, Jason Sheehan

**Affiliations:** 1 Department of Neurological Surgery, Mayo Clinic, Rochester, MN USA; 2 Department of Neurological Surgery, University of Virginia, P.O. Box 800212, Charlottesville, VA USA; 3 TheWalton Centre NHS Foundation Trust, Liverpool, UK; 4 Department of Neurosurgery, Koc University School of Medicine, Istanbul, Turkey; 5 Gamma Knife Center Cairo-Nasser Institute, Benha University, Benha, Egypt; 6 Division of Neurosurgery, Université de Sherbrooke, Centre de Recherche du CHUS, Sherbrooke, QC Canada; 7 Department of Neurosurgery, School of Medicine, Neurological Institute, Taipei Veteran General Hospital, Taipei, Taiwan; 8 Department of Radiation and Stereotactic Neurosurgery, Na Homolce Hospital, Prague, Czech Republic; 9 Department of Radiosurgery, Rúber International Hospital, Madrid, Spain; 10 Department of Neurosurgery, New York University, New York, NY USA; 11 Department of Radiation Oncology, New York University, New York, NY USA; 12 Department of Neurosurgery and Radiotherapy, Nehru Hospital, Postgraduate Institute of Medical Education and Research, Sector 12, Chandigarh, India; 13 Department of Radiology, Dominican Gamma Knife Centerand, CEDIMAT, Santo Domingo, Pepillo Salcedo Dominican Republic; 14 Department of Neurosurgery, University of Alberta, Edmonton, AB Canada; 15 Department of Neurosurgery, University of Miami Miller School of Medicine, Miami, FL USA; 16 Department of Neurosurgery, University of Pittsburgh, Pittsburgh, PA USA

**Keywords:** Meningioma, Elderly, Surveillance, Stereotactic radiosurgery

## Abstract

**Introduction:**

Management for elderly patients (> 65yo) with incidental meningiomas remains unclear. This study aims to characterize the functional and tumor outcomes of expectant and stereotactic radiosurgery (SRS) management of asymptomatic meningioma elderly patients.

**Methods:**

Using retrospectively collected data from 14 centers, SRS outcomes were compared to radiographic and clinical observation of asymptomatic meningiomas in elderly patients following propensity score matching.

**Results:**

Following propensity score matching, 114 patients were in each cohort. Tumor control was achieved at 97.37% in the SRS cohort, and no meningioma growth was seen 71.93% of the observation cohorts (*p* < 0.01; OR 14.44 [95% CI 4.27–48.78]). New neurological deficits developed in 1.39% of the SRS cohort but in none of the patients managed conservatively. 3.5% of patients underwent resection in the active surveillance matched cohort compared to 0.9% of patients in the SRS cohort (*p* = 0.063; OR 0.135 [95% CI 0.163–1.117]). The all-cause mortality rate was almost half in the SRS group (9.65%) compared to the observation group (18.42%) (*p* = 0.06; OR 0.47 [95% CI .22–1.03]).

**Conclusion:**

SRS achieves superior radiological tumor control compared to surveillance but with a slightly increased the risk of new SRS-related neurological deficits in elderly patients with asymptomatic meningiomas. Although SRS reduces meningioma progression, the need for of an open neurosurgical procedure and mortality were not significantly reduced. Furthermore, mortality in the observation group was not directly related to the meningioma in any of the patients.

## Introduction

Meningiomas are the most common benign intracranial tumors, and they arise from the meningothelial (arachnoid) cells. [27, 20] The aggregate incidence rate is 5—12 per 100,000 patients in the average population [27, 20], and steadily increases with advancing age to 25–30 per 100,000 in patients over 65, and 50 per 100,000 in patients over 85. [20, 29, 28, 5, 1] Approximately 39% of newly diagnosed meningiomas are asymptomatic and the prevalence is significantly higher in patients over 70 years old compared with younger patients (49.4% vs. 34%) [10, 13]. The overall increase in asymptomatic meningioma incidence corresponds with an increasing life expectancy and a growing pool of elderly patients with meningiomas, in addition to the increased availability and utilization of brain imaging for head injuries and other nonspecific neurological symptoms. Thus, an increasing number of elderly patients presenting with asymptomatic meningiomas is expected. [27, 1, 10, 13]

The management of asymptomatic meningiomas is subject to debate, particularly in elderly patients who are predisposed to comorbidities and frailty which influence management options. Evidence suggests that the majority of asymptomatic meningiomas do not enlarge [11, 6, 24, 8], and elderly patients are less likely to experience progression [11, 12]. However, 24–92% of moderately sized meningiomas demonstrate a linear increase in diameter. With growth, tumors particularly in skull base locations such as the cavernous sinus have demonstrated a high rate of new neurological signs or symptoms [24]. The most common chosen approach for meningiomas < 2 cm is observation. In small to moderate sized incidental meningiomas, change in volumetric assessments may vary occur in 18.8–88%; morbidity from surgical resection is higher in asymptomatic patients and may not justify the risk benefit ratio. Stereotactic radiosurgery is increasingly being used in the treatment of meningiomas, and due to the noninvasive nature, it is sometimes utilized for asymptomatic meningiomas. This International Multicenter Matched Cohort Analysis of Incidental Meningioma Progression During Active Surveillance or After Stereotactic Radiosurgery (IMPASSE) study [23] is a multicenter matched cohort analysis that aims to evaluate the efficiency and safety of SRS in the treatment of elderly patients with asymptomatic meningiomas.

## Methods

### Study population

The study included patients > 65 years old with incidental and asymptomatic meningiomas treated with SRS or managed conservatively from an international multicentric study where the detailed methodology is described [23]. In summary, baseline patient, tumor, and treatment variables as well as longitudinal follow-up data for patients diagnosed with incidental meningiomas were shared following SRS treatment by 14 centers in 10 nations and following active surveillance (observation cohort) by clinical and imaging monitoring from 18 hospitals in a regional health district. Comparable data was collected for the observation group of asymptomatic meningiomas managed by active clinical and radiologic surveillance (observation cohort) from an IRRF site (the University of Virginia) and the Walton Centre NHS Foundation Trust. The local institutional review board approved sharing anonymized data with the International Radiosurgery Research Foundation (IRRF) coordinating office. Due to the retrospective nature of this study, patient consent was waived.

Meningiomas were diagnosed based on imaging findings of extra-axial, dural-based lesions with homogenous contrast enhancement on T1-weigted brain MRI in the absence of past cancer history. Patients with symptomatic or multiple meningiomas were excluded from the study. Patients < 65 years old were excluded from this subanalysis. In both the SRS and observation groups, patients were followed by longitudinal clinical and neuroimaging assessments according to the respective institutional protocol. Tumor progression and time to progression were defined by the Response Assessment in Neuro-Oncology (RANO) criteria[9] within the longitudinal follow-up period. Tumor control was defined as a stable or regressed tumor on MRI at last follow up.

### Intervention

As per a consensus definition, SRS was performed in a single session using the Gamma Knife (Elekta AB, Stockholm, Sweden), and MRI and/ or CT with contrast were used for stereotactic targeting in a multi-isocentric approach. The local clinical team decided the SRS technique and doses according to local protocols and available radiosurgical technology.

### Outcomes

The primary outcome of the study is local control defined as stable or regressed tumor on neuroimaging in accordance with the RANO criteria[9] where a tumor is defined as stable if its volume changed by less than 25% and regressed if decreased by ≥ 25 of the baseline volume. Secondary outcomes were tumor progression, development of new neurological deficit, KPS score, and all-cause mortality. A new neurological deficit was defined as a cranial nerve deficit, sensory disturbance, motor dysfunction, or change in global status attributable to the tumor and not present at time of initial presentation or pre-treatment in the SRS cohort.

### Statistical analysis

All statistical analyses were conducted using Stata (StataCorp. 2021. Stata Statistical Software: Release 17. College Station, TX: StataCorp LLC). Baseline characteristics and outcomes were compared between the SRS and the observation cohorts. Shapiro-Francia tests were used to evaluate data normality. Continuous variables were compared by student t-tests, and categorical variables were compared using Pearson χ ^2^.

Patient age, tumor volume on presentation and length of follow-up have been demonstrated to impact the natural history of meningiomas. As random selection for treatment group is not appropriate, the SRS and observation groups were matched in a 1:1 ratio without replacement using propensity scores derived from patient age, tumor volume and location, and duration of neuroimaging follow up using the PSMATCH2 package for Stata 17 to limit confounding results. Adequate balance for the matched covariates was considered an absolute standardized difference < 0.1 between the 2 cohorts. Advanced age has been shown to be related to the natural history of meningiomas; outcomes were stratified by elderly age groups. Univariate comparisons of the unmatched and matched cohorts were performed for outcome measures using binary logistic regression analysis. Statistical significance was defined as *p*-value < 0.05, and all tests were 2-tailed. Missing data were not imputed.

## Results

### Unmatched patient and tumor attributes

In the unmatched cohorts, data was collected on 193 SRS patients and 166 patients managed with close observation (Table 1). The mean age of the SRS group was 72.84 (± 5.9) years and the observation group 73.13 (± 5.72) years (*p* = 0.65). Median initial Karnofsky Performance Score (KPS) was 100 (range: 60–100) and 90 (range: 50–100) in the observation group (*p* < 0.01). Mean meningioma volume of the SRS and observation groups were 5.14(± 4.44) and 4.81(± 6.46) cm3, respectively (*p* = 0.56). Mean radiologic follow-up durations for the SRS and observation cohorts were 50.9(± 40.67) and 32.9(± 32.9) months respectively (*p* < 0.01). Mean clinical follow-up durations were 51.63(± 40.67) and 32.90(23.69) months respectively (*p* < 0.01). The mean marginal dose to the tumor was 12.94 (± 1.65) Gy and mean maximum dose was 25.62(± 4.11) in the SRS cohort.

**Table 1:** Unmatched cohort baseline demographics

Characteristic	Total (*n* = 359)	SRS (*n* = 193)	Observation (*n* = 166)	*p*-value
Age, mean yr (SD; range)	72.9 (5.9; 65–102)	72.84 (6.08; 65–102)	73.13 (5.72; 65–90)	0.6533
Male, *n* (%)	97 (27)	52 (26.9)	45 (27.1)	0.972
Baseline KPS, median (range)	90 (50–100)	100 (60–100)	90 (50–100)	** < 0.01**
Diameter, mean mm (SD)	20.69 (9.44)	20.47 (9.37)	20.94 (9.53)	0.6439
Volume, mean cm^3^ (SD)	4.98 (5.46)	5.14 (4.44)	4.81 (6.46)	0.5598
Laterality, *n* (%)				0.039
Right	174/357 (48.74)	83/357 (23.25)	91 (25.49)	
Left	161/357 (45.9)	92/357 (25.77)	69/357 (19.33)	
Midline	22/357 (6.16)	16/357 (4.48)	6/357 (1.68)	
Location, n (%)				** < 0.01**
Skull base	143 (39.83)	93 (48.18)	50 (30.12)	
Convexity	84 (23.39)	29 (8.07)	55 (15.32)	
Other	127 (55.7)	67 (58.77)	60 (52.63)	
Margin dose, mean Gy (SD)		12.94 (1.65)		
Maximum dose, mean Gy (SD)		25.62 (4.11)		
Isocenters, median (IQR)		12 (7–18)		
Treatment volume, mean cm^3^ (SD)		6.29 (5.08)		
Imaging FU, mean in months (SD)	42.58 (34.79)	50.9 (40.3)	32.90 (32.903)	** < 0.01**
Clinical FU, mean in months (SD)	42.96 (35.1)	51.63 (40.67)	32.90 (23.69)	** < 0.01**

*KPS* Karnofsky Performance Scale; *FU* Follow up

### Matched patient and tumor attributes

Following propensity score matching for patient age, tumor location, tumor volume, and duration of radiologic follow-up, 114 patients remained in each cohort (Table 2). The mean age was 73.11(± 6.52) years in the SRS cohort and 72.53(± 5.48) years in the observation group (*p* = 0.46). Median baseline KPS score was 100 (range: 70–100) in the SRS and 90 (range: 50–100) in the observation cohorts (*p* < 0.01). Mean tumor volume was 5.13(± 4.41) cm^3^ in the SRS and 4.93(± 6.54) cm^3^ in the observation groups (*p* = 0.78). Radiologic and clinical follow-up durations were not statistically different between cohorts.

**Table 2:** Matched cohort baseline demographics

Characteristic	Total (*n* = 228)	SRS (*n* = 114)	Observation (*n* = 114)	*p*-value
Age, mean yr (SD; range)	72.82 (6.01; 65–102)	73.11 (6.52; 65–102)	72.53 (5.48; 65–89)	0.46
Male, n (%)	62 (27.2)	32 (28.1)	30 (26.3)	0.76
Baseline KPS, median (range)	90 (50–100)	100 (70–100)	90 (50–100)	** < 0.01**
Diameter, mean mm (SD)	21.13 (9.29)	20.88 (8.75)	21.37 (9.80)	0.69
Volume, mean cm^3^ (SD)	5.03 (5.56)	5.13 (4.41)	4.93 (6.54)	0.78
Laterality, *n* (%)				0.78
Right	113/227 (49.65)	55/227 (48.23)	58/227 (50.88)	
Left	101/227 (44.29)	50/227 (43.86)	51/227 (44.74)	
Midline	13/227 (5.7)	8/227 (7.02)	5/227 (4.39)	
Location, *n* (%)				** < 0.01**
Skull base	94 (42.54)	47 (41.23)	47 (41.23)	
Convexity	58 (25.44)	29 (25.44)	29 (25.44)	
Other	74 (16.23)	37 (32.46)	37 (32.46)	
Margin dose, mean Gy (SD)		12.89 (1.35)		
Maximum dose, mean Gy (SD)		25.14 (3.72)		
Isocenters, median (IQR)		12 (7–18)		
Treatment volume, mean cm^3^ (SD)		6.18 (4.7)		
Imaging FU, mean in months (SD)	42.87 (32.08)	46.43 (37.29)	39.31 (25.52)	0.09
Clinical FU, mean in months (SD)	43.26 (32.54)	47.21 (38.01)	39.31 (25.52)	0.06

*KPS* Karnofsky Performance Scale; *FU* Follow up

### Radiologic and neurologic outcomes for unmatched cohorts

In the unmatched cohorts, tumor control was achieved in 98.45% of patients who received SRS. In the observation cohort tumors were stable and did not grow in 71.1% of patients (*p* < 0.01; OR 11.48 [95% CI 5.68–62.2]) (Table 3). Tumor regression was observed in 42.49% of patients following SRS treatment and in 1.2% of patients following observation (*p* < 0.01; OR 60.58 [95% CI 14.59–251.42]). Tumor progression was less frequent in the SRS treated cohort (1.55%) compared to the observation cohort (22.89%) (*p* < 0.01; OR 0.05 [95% CI 0.016–0.18]).

**Table 3:** Unmatched cohort outcomes

Characteristic	SRS (*n* = 193)	Observation (*n* = 166)	OR (95% CI)	*p*-value
Total				
Tumor control, n (%)	190 (98.45)	128 (71.1)	11.48 (5.68–62.2)	** < 0.01**
Tumor regression, n (%)	82 (42.49)	2 (1.2)	60.58 (14.59–251.42)	** < 0.01**
Tumor progression, n (%)	3 (1.55)	38 (22.89)	0.05 (0.016–0.18)	** < 0.01**
New neurological deficit, n (%)	3 (1.55)	0	1	**−**
KPS at LFU, median (range)	100 (40–100)	90 (50–100)	−	** < 0.01**
All-cause mortality, n (%)	20 (10.36)	33 (19.88)	0.46 (0.26–0.85)	**0.01**
65–75 years	SRS *n* = 129	Observation *n* = 105	OR (95% CI)	*p*-value
Tumor control, *n* (%)	127 (98.45)	78 (74.29)	21.98 (5.09–95)	** < 0.01**
Tumor regression, *n* (%)	48 (37.21)	1(0.95)	61.63 (8.33–456.05)	** < 0.01**
Tumor progression, *n* (%)	2 (1.55)	27 (25.71)	0.05 (0.01–0.19)	** < 0.01**
New neurological deficit, *n* (%)	2 (1.55)	0	1	**−**
KPS at LFU, median (range)	90 (40–100)	90 (50–100)	−	**0.14**
All-cause mortality, *n* (%)	6 (4.65)	11 (10.48)	0.42 (0.15–1.17)	**0.09**
76–85 years	SRS *n* = 60	Observation *n* = 57	OR (95% CI)	*p*-value
Tumor control, *n* (%)	59 (98.34)	46 (80.7)	14.11 (1.76–113.28)	**0.013**
Tumor regression, *n* (%)	33 (55)	0	1	**−**
Tumor progression, *n* (%)	1 (1.67)	11 (19.29)	0.07 (0.01–0.57)	**0.013**
New neurological deficit, *n* (%)	1 (1.67)	0	1	**−**
KPS at LFU, median (range)	100 (80–100)	90 (50–100)	−	** < 0.01**
All-cause mortality, *n* (%)	14 (23.34)	19 (33.34)	0.61 (0.27–1.37)	**0.23**
> 85 years	SRS *n* = 4	Observation *n* = 4	OR (95% CI)	*p*-value
Tumor control, *n* (%)	4 (100)	4 (100)	−	**−**
Tumor regression, *n* (%)	1 (25)	1 (25)	−	−
Tumor progression, *n* (%)	0	0	−	−
New neurological deficit, *n* (%)	0	0	−	−
KPS at LFU, median (range)	85 (70–100)	90 (70–90)	−	**1**
All-cause mortality, *n* (%)	0	3 (75)	−	−

*KPS* Karnofsky Performance Scale; *LFU* LAst follow up

New neurological deficit developed in 1.55% of the SRS cohort compared to none of the patients in the observation cohort; among the patients with new neurological deficit, none of the patients had tumor progression and the deficit was due to the SRS treatment. The most common tumor locations in patients who developed new deficits were skull base, parasagittal, and falx. 4.2% of patients in the observation cohort required resection compared to 1% in the SRS cohort, however this was not statistically significant (*p* = 0.07; OR 0.237 [95% CI 0.048–1.161]). The observed all-cause mortality rate was 10.36% in the SRS group compared to 19.88% in the observation cohort (*p* = 0.01; OR 0.46 [95% CI 0.26–0.85]), however this does not infer tumor-related mortality. Outcomes did not greatly differ by age groupings of 65–75 years; 75–85 years; and 85 + years in the unmatched cohort (Table 3).

### Radiologic and neurologic outcomes for matched cohorts

In the matched cohort (Table 4), tumor control was 97.37% after SRS intervention, whilst 71.93% of observed meningioma did not grow (71.93%) (*p* < 0.01; OR 14.44 [95% CI 4.27–48.78]). Tumor regression was found in 37.72% of the SRS cohort and in none of the patients managed conservatively. Tumor progression was found in 1.75% of the SRS and in 28.07% of the observation cohorts (*p* < 0.01; OR 0.046 [95% CI 0.01–0.196]).

**Table 4:** Matched cohort outcomes

Characteristic	SRS (*n* = 114)	Observation (*n* = 114)	OR (95% CI)	*p*-value
Tumor control, *n* (%)	111 (97.37)	82 (71.93)	14.44(4.27–48.78)	** < 0.01**
Tumor regression, *n* (%)	43 (37.72)	0	1	−
Tumor progression, *n* (%)	2 (1.39)	32 (22.22)	.046 (0.01–0.196)	** < 0.01**
New neurological deficit, *n* (%)	2 (1.39)	0	1	**−**
KPS at LFU, mean (SD)	100 (70–100)	90 (50–100)	−	** < 0.01**
All-cause mortality, *n* (%)	11 (9.65)	21 (18.42)	0.47 (.22–1.03)	0.06
65–75 years	SRS *n* = 75	Observation *n* = 75	OR (95% CI)	*p*-value
Tumor control, *n* (%)	73 (97.34)	52 (69.32)	16.14 (3.65–71.49)	** < 0.01**
Tumor regression, *n* (%)	26 (34.67)	0	1	**−**
Tumor progression, *n* (%)	1 (1.34)	23 (30.67)	0.03 (0.01–0.23)	** < 0.01**
New neurological deficit, *n* (%)	2 (2.67)	0	1	−
KPS at LFU, median (range)	100 (70–100)	90 (50–100)	−	0.07
All-cause mortality, *n* (%)	2 (2.67)	7 (9.34)	0.27 (0.05–1.33)	0.11
76–85 years	SRS *n* = 35	Observation *n* = 38	OR (95% CI)	*p*-value
Tumor control, *n* (%)	34 (97.14)	26 (68.42)	10.56 (1.26–88.31)	**0.03**
Tumor regression, *n* (%)	16 (45.71)	0	1	−
Tumor progression, *n* (%)	1 (2.86)	9 (23.68)	0.09 (0.01–0.79)	**0.03**
New neurological deficit, *n* (%)	0	0	−	−
KPS at LFU, median (range)	100 (80–100)	90 (50–100)	−	** < 0.01**
All-cause mortality, *n* (%)	9 (25.71)	13 (34.21)	0.67 (0.24–1.83)	0.43
> 85 years	SRS *n* = 4	Observation *n* = 1	OR (95% CI)	*p*-value
Tumor control, *n* (%)	4 (100)	1 (100)	−	−
Tumor regression, *n* (%)	1 (25)	0	−	−
Tumor progression, *n* (%)	0	0	−	−
New neurological deficit, *n* (%)	0	0	−	−
KPS at LFU, median (range)	85 (70–100)	90	−	−
All-cause mortality, *n* (%)	0	1 (100)	−	−

*KPS* Karnofsky Performance Scale; *LFU* Last follow up

Similar to the unmatched cohort, new neurological deficits developed in 1.39% of the SRS cohort but in none of the patients managed conservatively. In those with new neurological deficits, none of these patients exhibited tumor progression and the deficit was a side effect of the SRS treatment. The tumor locations associated with new deficits were the same in the matched cohort. In the observation cohort, 3.5% of patients underwent resection compared to 0.9% of patients in the SRS cohort, however this was not statistically significant (*p* = 0.063; OR 0.135 [95% CI 0.163–1.117]).

In the observation cohort, radiological progression was reported in seven patients (median age: 67; range: 65–73). Gross total resection was achieved in five patients with no surgical or medical complications. Meningioma was grade I in four patients none of which had recurrence at last follow- up from 30–56 months. Meningioma was grade II in one patient who experienced recurrence at 5 months and was treated with salvage radiotherapy after which there was no further growth. One patient was managed symptomatically (seizure control) and elected not to undergo resection. One patient underwent fractionated stereotactic radiotherapy; Early Common Terminology Criteria for Adverse Events (CTCAE) toxicity included nausea grade 1 and fatigue grade 2. Late CTCAE toxicity included trigeminal neuralgia-grade 2. Duration of follow-up was 8 months. At that point, the patient was still troubled by her neuralgia.

In the SRS cohort, two patients underwent further surgical resection, one of which had no surgical morbidity or mortality, and the other was lost to follow-up.

The all-cause mortality rate was almost half in the SRS group (9.65%) compared to the observation group (18.42%), however this was not statistically significant (*p* = 0.06; OR 0.47 [95% CI 0.22–1.03]). Outcomes were comparable by age groupings of 65–75 years; 75–85 years; and 85 + years in the matched cohort (Table 4).

## Discussion

This matched cohort multicenter analysis evaluated the clinical and radiological results of 359 elderly patients over 65 years of age diagnosed with asymptomatic meningiomas managed with either SRS (*n* = 193) or active surveillance (*n* = 166). In a matched cohort analysis, which included 114 patients from each cohort, tumor progression was noted in 28% of patients in the observation group but in only 1.75% of patients that underwent SRS. Upfront SRS treatment of elderly patients for asymptomatic meningiomas yielded significantly better tumor control rates. While SRS did result in a low rate of neurological symptoms, it allowed elderly patients a chance of avoiding a craniotomy and tumor resection, however the reduction was not statistically significant. The all-cause mortality rate was lower in the group treated with SRS.

Convexity meningiomas are the most common location in the elderly population which is reflected by the findings in the present study [25]. Convexity meningiomas tend to develop symptoms at a later time interval compared to nonconvexity meningiomas which validates the higher incidence in this cohort [25]. Cognitive impairment is a frequently underrecognized presenting symptom of meningiomas that are otherwise labeled asymptomatic especially in elderly patients who are prone to neurocognitive decline from an alternative pathophysiology. It is important to make this distinction and maintain a high index of suspicion for otherwise large asymptomatic meningiomas as a possible etiology for any cognitive deficits given that resection may reverse these symptoms [6, 15, 26, 4].

In a recent study, it was identified that the indication for neuroimaging resulting in the diagnosis of incidental meningioma was statistically different in elderly patients compared to younger patients; cerebrovascular incidents and cognitive changes were the most common indications in the elderly compared to headaches in younger patients [16]. Moreover, it was noted that 10.3% more elderly patients were undergoing neuroimaging for brain metastasis in recent years compared to a decade ago [16]. It is important to consider dural metastasis as a differential diagnosis, especially in the elderly as meningiomas are increasingly being diagnosed radiologically with the conservative management trends and frequent history of malignancy in this patient population [18]. Epidemiological studies demonstrate an increase in incidental radiological diagnoses of meningiomas but unchanged rates of histopathological diagnoses, which underscores these trends to conservative management [6, 2].

Nonoperative management of asymptomatic meningiomas is the mainstay, particularly in the elderly population which is predisposed to frailty and comorbidities [6, 2]. While current guidelines recommend radiological and clinical surveillance of incidental meningiomas followed by SRS in the event of radiological and/or clinical progression, the present study demonstrates multicenter data suggesting that SRS affords local tumor control as per RANO definitions of stability or regression without significant risk of morbidity or mortality in this frail population at an average of 33 months follow up. In both the unmatched and matched analysis, conversative management of meningiomas in the elderly led to a marginally higher rate of craniotomy and tumor resection. Although prolonged further follow up would be required to validate the durability and safety of SRS, this may be less relevant for the elderly patient. Santacroce et al. described WHO grade I meningioma 5- and 10-year PFS of 95.2% and 88.6%, respectively, also with insignificant morbidity in over 3,700 meningiomas treated with SRS, validating the results in the present study [22]. Reuß et al. and Hasegawa et al. reported similar rates of PFS and morbidity in studies stratified to an elderly patient cohort, with patients > 70 years having a slightly higher risk for SRS toxicity [21, 7]. In the present study, tumor and functional outcomes including new neurological deficits did not significantly differ from 65 to over 85 years of age. However, the sample size was reduced in older age groups limiting the generalizability of present results. Furthermore, the duration of follow may be inadequate to accurately reflect meningioma progression, development of symptoms, and eventual need for resection. Reassessment of outcomes using a larger dataset is validated.

The natural history of meningiomas in elderly patients demonstrates a slightly lower rate of progression compared to younger patients [6]. However this may be attributable to shorter follow-up duration. Niiro et al. reported a 35% progression rate in an average tumor size 30.9 mm (median 30 mm, range 18–60 mm) and a mean follow up of 32.1 months (median 30 months, range 10–88 months), comparable with the data in the present study [17]. In their series, 35.7% of meningiomas that progressed became symptomatic, with one of case of mortality attributable to meningioma progression [17]. The SRS group had a higher median KPS than the observation group, which suggest there is an element of selection bias for giving radiosurgery as an intervention. Due to data limitations is it not possible to assign mortality to the meningioma or SRS. It would be reasonable to study the effect of potential SRS adverse events on mortality in long term follow up particularly in a matched cohort. Elderly patients with small, asymptomatic lesions with low-risk radiological features are unlikely to develop rapid growth. Additionally, slow growth, if any, may not reach a clinically significant volume within the patient’s lifetime [14]. However, as shown by the UCSF team, patients with meningiomas particularly in the cavernous sinus and petroclival areas are at increased risk for development of neurological signs and symptoms when left untreated [24]. In the current study, the need for resection was not significantly higher in the observation cohort.

The initial IMPASSE study of 1,115 patients revealed significantly favorable radiological outcomes in the SRS cohort, but similar rates of neurological deficits compared to the observed cohort [23]. The present subanalysis of only elderly patients in that cohort revealed similarly favorable rates of radiological tumor control compared to the observation cohort. However, with a short follow up of less than 3 years, no patients who were observed developed new neurological deficits. However, in patients who had SRS, the risk persisted in about 1.3% of patients. Although SRS achieves higher rates of tumor control, the risk of radiological and/or clinical progression in this study and external literature persists for a considerably longer period of time and warrants longer term follow up studies.

## Limitations

Intrinsic to the methodology of a retrospective analysis, the study design subjects the results to bias and confounding factors in the analysis. Decisions for SRS or active surveillance could not be discerned based on the data available and may be a source of bias. Progression free survival could not be analyzed due to insufficient data. Malignant transformation was not documented. WHO grading may be miscalculated by radiological interpretation and lack of histopathology. Radiological follow up was not completed at the same time intervals or for the same average duration which may conceal to undocumented progression. Unfortunately, tumor related mortality was not discernable from all-cause mortality from the available data and could not be differentiated between the patient cohorts. While subgroup analyses of tumors by location would be of interest to clinicians and patients, the current study had insufficient power to permit meaningful subgroup analyses by tumor location.

## Conclusion

SRS demonstrates enhanced radiological tumor control over surveillance in elderly patients with asymptomatic meningiomas, albeit with a slightly elevated risk of new SRS-related neurological deficits. While SRS diminishes meningioma progression, it does not significantly reduce the necessity for an open neurosurgical procedure or the rate of tumor associated mortality. Moreover, mortality within the observation group was unrelated to meningioma across all patients. SRS may be safer for elderly patients with fewer comorbidities, however life expectancy and the potential need for resection in the future should also be taken into consideration on a per patient basis during shared decision making.

## Data Availability

No datasets were generated or analysed during the current study.

## Abbreviations

SRS: Stereotactic radiosurgery
IRRF: International Radiosurgery Research Foundation
RANO: Response Assessment in Neuro-Oncology
KPS: Karnofsky Performance Score
